# Draft Genome Sequence of *Pseudomonas aeruginosa* KF702 (NBRC 110665), a Polychlorinated Biphenyl-Degrading Bacterium Isolated from Biphenyl-Contaminated Soil

**DOI:** 10.1128/genomeA.00517-15

**Published:** 2015-05-21

**Authors:** Hidehiko Fujihara, Atsushi Yamazoe, Akira Hosoyama, Hikaru Suenaga, Nobutada Kimura, Jun Hirose, Takahito Watanabe, Taiki Futagami, Masatoshi Goto, Kensuke Furukawa

**Affiliations:** a Department of Food and Nutrition, Beppu University, Beppu, Japan; b Biological Resource Center, National Institute of Technology and Evaluation (NITE), Tokyo, Japan; c Bioproduction Research Institute, National Institute of Advanced Industrial Science and Technology (AIST), Tsukuba, Japan; d Faculty of Engineering, Department of Applied Chemistry, University of Miyazaki, Miyazaki, Japan; e Research Institute for Sustainable Humanosphere, Kyoto University, Kyoto, Japan; f Education and Research Center for Fermentation Studies, Faculty of Agriculture, Kagoshima University, Kagoshima, Japan; g Laboratory of Future Creation Microbiology, Faculty of Agriculture, Kyushu University, Fukuoka, Japan

## Abstract

*Pseudomonas aeruginosa* KF702 (NBRC 110665) utilizes biphenyl as a sole source of carbon and degrades polychlorinated biphenyls (PCBs). Here, we report the 7,167,540-bp draft genome sequence of KF702, which contains 6,714 coding sequences and a 65.8 mol% G+C content. The strain possesses genes for biphenyl catabolism and other genes that mediate degradation of various aromatic compounds.

## GENOME ANNOUNCEMENT

Polychlorinated biphenyls (PCBs) have been recognized as significant environmental pollutants for a long time. Biphenyl-utilizing bacteria cometabolize certain PCBs into chlorobenzoic acids through oxidation by biphenyl-catabolic enzymes. However, the biodegradabilities of PCBs are highly dependent on the chlorine substitutions, i.e., the number and position of substituted chlorines (1). It is also evident that the degradation capabilities of PCBs are different among the biphenyl-utilizing strains. The biphenyl catabolic *bph* genes were first cloned from *P. pseudoalcaligenes* KF707 (2). Since then, a number of PCB-degrading bacteria have been identified, including both Gram-negative and Gram-positive bacteria (3). Some strains possess very similar, if not identical, *bph* genes, while others possess diversified *bph* genes (4). The purpose of this study was to explore how *bph* genes are organized, transferred, and rearranged by sequencing the genomes of various PCB degraders isolated from the same site. We isolated 14 PCB-degrading bacterial strains (KF strains) including *Pseudomonas aeruginosa* KF702 from the same biphenyl-contaminated soil in Kitakyushu, Japan (4).

Whole-genome shotgun sequencing of *P. aeruginosa* KF702 was performed by the National Institute of Technology and Evaluation (NITE) using a combination of shotgun sequencing on a 454 Roche GS FLX+ system (Roche) and paired-end sequencing on a HiSeq sequencing system (Illumina). The Newbler software package (version 2.6, Roche) was used for the genome assembly. The draft genome was composed of 91 contigs (>701 bp), totaling 7,167,540 bases, with a G+C content of 65.8 mol%. The *N*_50_ contig size and the largest contig size were 210,619 bp and 464,066 bp, respectively.

Rapid genome annotation using the RAST annotation server (5) described 6,714 coding sequences (CDSs), 38 tRNA sequences, and three rRNA sequences. The coding sequences were classified into 573 subsystems, including cofactors, vitamins, prosthetic groups, and pigments (*n* = 335 CDSs); phages, prophages, transposable elements, and plasmids (*n* = 69 CDSs); iron acquisition and metabolism (*n* = 148 CDSs); motility and chemotaxis (*n* = 114 CDSs); nitrogen metabolism (*n* = 88 CDSs); metabolism of aromatic compounds (*n* = 164 CDSs); and carbohydrates (*n* = 540 CDSs). Comparison of *P. aeruginosa* KF702 with other *Pseudomonas* strains within the RAST server database identified *P. aeruginosa* 19BR (taxonomy identification no. 1051003.3) as its closest neighbor, with a score of 538, followed by *P. aeruginosa* LESB58 (taxonomy identification no. 557722.3) as the 27th closest neighbor, with a score of 242. *P. aeruginosa* 19BR is known as a multidrug- and polymyxin-resistant strain (6).

Functional annotations were compared with the Kyoto Encyclopedia of Genes and Genomes (KEGG) (7). Strain KF702 possessed the *bph* genes cluster very similar to those of *P. pseudoalcaligenes* KF707 (8), entire benzoate- and salicylate-degradative genes via the hydroxylation pathways, and some genes of the protocatechuate-gentisate and cyclohexanone degradation.

### Nucleotide sequence accession numbers.

This draft genome sequence has been deposited at DDBJ/EMBL/GenBank under the accession numbers BBQK01000001 to BBQK01000091.
